# Systemic Low-Frequency Oscillations in BOLD Signal Vary with Tissue Type

**DOI:** 10.3389/fnins.2016.00313

**Published:** 2016-06-30

**Authors:** Yunjie Tong, Lia M. Hocke, Kimberly P. Lindsey, Sinem B. Erdoğan, Gordana Vitaliano, Carolyn E. Caine, Blaise deB. Frederick

**Affiliations:** ^1^McLean Imaging Center, McLean HospitalBelmont, MA, USA; ^2^Department of Psychiatry, Harvard University Medical SchoolBoston, MA, USA; ^3^Department of Radiology, University of CalgaryCalgary, AB, Canada

**Keywords:** BOLD, vascular density, amplitude of low-frequency fluctuation, low frequency oscillation, resting state fMRI

## Abstract

Blood-oxygen-level dependent (BOLD) signals are widely used in functional magnetic resonance imaging (fMRI) as a proxy measure of brain activation. However, because these signals are blood-related, they are also influenced by other physiological processes. This is especially true in resting state fMRI, during which no experimental stimulation occurs. Previous studies have found that the amplitude of resting state BOLD is closely related to regional vascular density. In this study, we investigated how some of the temporal fluctuations of the BOLD signal also possibly relate to regional vascular density. We began by identifying the blood-bound systemic low-frequency oscillation (sLFO). We then assessed the distribution of all voxels based on their correlations with this sLFO. We found that sLFO signals are widely present in resting state BOLD signals and that the proportion of these sLFOs in each voxel correlates with different tissue types, which vary significantly in underlying vascular density. These results deepen our understanding of the BOLD signal and suggest new imaging biomarkers based on fMRI data, such as amplitude of low-frequency fluctuation (ALFF) and sLFO, a combination of both, for assessing vascular density.

## Introduction

Blood-oxygen-level dependent (BOLD) contrast is the primary signal used in functional MRI (fMRI) studies of brain function. However, BOLD is not a direct measure of neuronal activity, but rather a blood-related composite signal that reflects changes in blood flow, volume, and oxygenation (Buxton, 2002). Thus, although neuronal activation can induce these changes through neurovascular coupling (Liu, 2013), BOLD signal changes can arise from any processes that affect the blood including nonneuronal and systemic physiological changes. Many aspects of the BOLD LFO—including its amplitude, frequency distribution, and temporal characteristics—have been examined independently in attempts to parse the neuronal and physiological contributions to the signal (Birn et al., 2008; Chang et al., 2009; Frederick et al., 2012; Murphy et al., 2013).

The amplitude of low-frequency fluctuation (ALFF) of BOLD has been studied extensively (Zang et al., 2007; Zuo et al., 2010; Kannurpatti et al., 2012; Vigneau-Roy et al., 2014), and it has been shown that ALFF is higher in gray matter (GM) than in white matter (WM) (Biswal et al., 1995; Cordes et al., 2001; Zang et al., 2007; Yan et al., 2009; Zuo et al., 2010). Studies found the highest ALFF in posterior structures along the midline (Zang et al., 2007; Zou et al., 2008, 2009). Recently, Vigneau-Roy et al. (2014) found a close relationship between regional variations in vascular density and ALFF of resting state BOLD signal. Based on this finding, they concluded that resting state BOLD signals are closely associated with the anatomical vasculature, and suggested calibration of resting state data using the venous structure (Barth and Norris, 2007). The result implied that ALFF could be a potential biomarker to assess vascular integrity.

In this study, we explored an additional vascular biomarker in the temporal fluctuations of LFO (0.01~0.15 Hz) in the resting state BOLD fMRI signal. Previously, we found that a significant portion of the LFO in resting state BOLD data can be attributed to blood circulation. Using this BOLD LFO signal and its temporal shifts, dynamic patterns resembling cerebral blood flow were derived from resting state data (Tong and Frederick, 2010, 2014). This finding indicates the tight connection between systemic LFO (sLFO) and the global blood circulation. We have studied the temporal delays of this sLFO in BOLD extensively. However, its correlation strength with BOLD has not been evaluated previously. In the present study, we wanted to study the relationship between the correlation strength (between sLFO and BOLD) in each voxel and the voxel's underlying vascular density. Since vascular density is hard to assess using the current MR methods, especially vascular density within the same tissue type (e.g., GM), we use three easily identifiable tissue types (i.e., WM, GM, and big blood vessels) to represent increasing vascular contents. We hypothesized that the higher the correlation between sLFO and BOLD, the greater the contribution of systemic blood-borne signal fluctuations to that voxel and, thus, the more likely this voxel would be found in the tissue with high vascular density region (e.g., GM). To test this hypothesis, we collected resting state fMRI data and then completed the following steps for each participant. First, we segmented each scanned brain into three tissue types, which have very different vascular densities: white matter (WM) (lowest), gray matter (GM) (intermediate) and vasculature (VA) (highest). We then calculated the voxel-wise peak cross-correlation map (3D), in which the value in each voxel represents the maximum correlation coefficient value between the BOLD signal and the optimally shifted sLFO signal. Finally, we explored the spatial distribution of these voxels in the three brain tissues according to their maximum correlation value. As a comparison, we also calculated ALFF for each participant's resting state data.

## Materials and methods

### Protocol

fMRI resting state studies were conducted in 8 healthy participants (average 33 ± 12, years). In the resting state studies, participants were asked to lie quietly in the scanner with their eyes open and view a gray screen with a fixation point in the center. The resting state scans lasted 6 min. All subjects provided informed, written consent. McLean Hospital Institutional Review Board approved the research protocol, which was conducted in accordance with the ethical principles of the Belmont Report.

All MR data were acquired on a Siemens TIM Trio 3T scanner (Siemens Medical Systems, Malvern, PA) using a 32-channel phased array head matrix coil. After scans for localization and automated alignment, multiecho multiplanar rapidly acquired gradient-echo (ME-MPRAGE) structural images were acquired with the following parameters (*TR* = 2530 ms, *TE* = 3.31,6.99,8.85,10.71 ms, *TI* = 1100 ms, slices = 128, matrix = 256 × 256, flip angle = 7°, resolution = 1.0 × 1.0 × 1.33 mm, 2 × GRAPPA, total acquisition time 4:32). Multiband EPI (University of Minnesota sequence cmrr_mbep2d_bold R010) (Moeller et al., 2010) data was obtained with parameters approximating that of the the Human Connectome Project (Van Essen et al., 2013) fMRI protocol: TR/TE = 720/32 ms, flip angle 66 degrees, matrix = 86 × 86 on a 212 × 212 mm^2^ FOV, posterior to anterior phase encode, multiband factor = 8, 64 2.5 mm slices with no gap parallel to the AC-PC (anterior commissure–posterior commissure) line extending down from the top of the brain. An MRI-compatible optical NIRS probe (1.5 cm separation between collection and illumination fibers) was placed over the tip of the left middle finger. NIRS data was recorded continuously before, during, and after the resting state fMRI acquisition with an ISS Imagent (ISS, Inc., Champaign, IL) at 690 and 830 nm with 25 Hz acquisition rate. For seven out of eight participants, 3D phase contrast magnetic resonance angiography (MRA) data were acquired (TR/TE = 40.95/6.21 ms, flip angle 15 degrees, matrix = 200 × 150 mm in plane (0.39 mm in-plane resolution), GRAPPA = 2, 160 0.9 mm slices with 0.18 mm gap, velocity encoded at 30 and 75 cm/s) to assess the cerebral vasculature.

### Preprocessing

For each participant, the standard fMRI preprocessing steps, including brain extraction, motion correction, slice-time correction and smoothing (3 mm), were applied to the original BOLD signals (using FEAT v6.00 of FSL 5.0; Jenkinson et al., 2012). We then applied a Fourier domain bandpass filter (zero-phase digital filter function “filtfilt,” which uses a third order Butterworth filter with cut-off frequencies: 0.01~0.15 Hz) in MATLAB (The Mathworks, Natick, MA) on all the resulting data to remove the high-frequency physiological signals of respiration and cardiac pulsation from the BOLD data. The resulting data were subsequently used to generate the maps described below.

We applied pipelines from FAST (Zhang et al., 2001) (FSL) on each participant's anatomical brain to segment WM, GM, and cerebrospinal fluid (CSF) regions. These segmented regions, the MRA scans and each participant's own resting state fMRI volumes were registered to the MNI152 standard brain. VA of each subject was derived from each subject's thresholded MRA scan. The threshold of 50 (applied to all the subjects) was an empirical value decided based on the results of all the subjects and the scan parameters, which rendered explicit VA maps for all the subjects.

**Figure 1:**
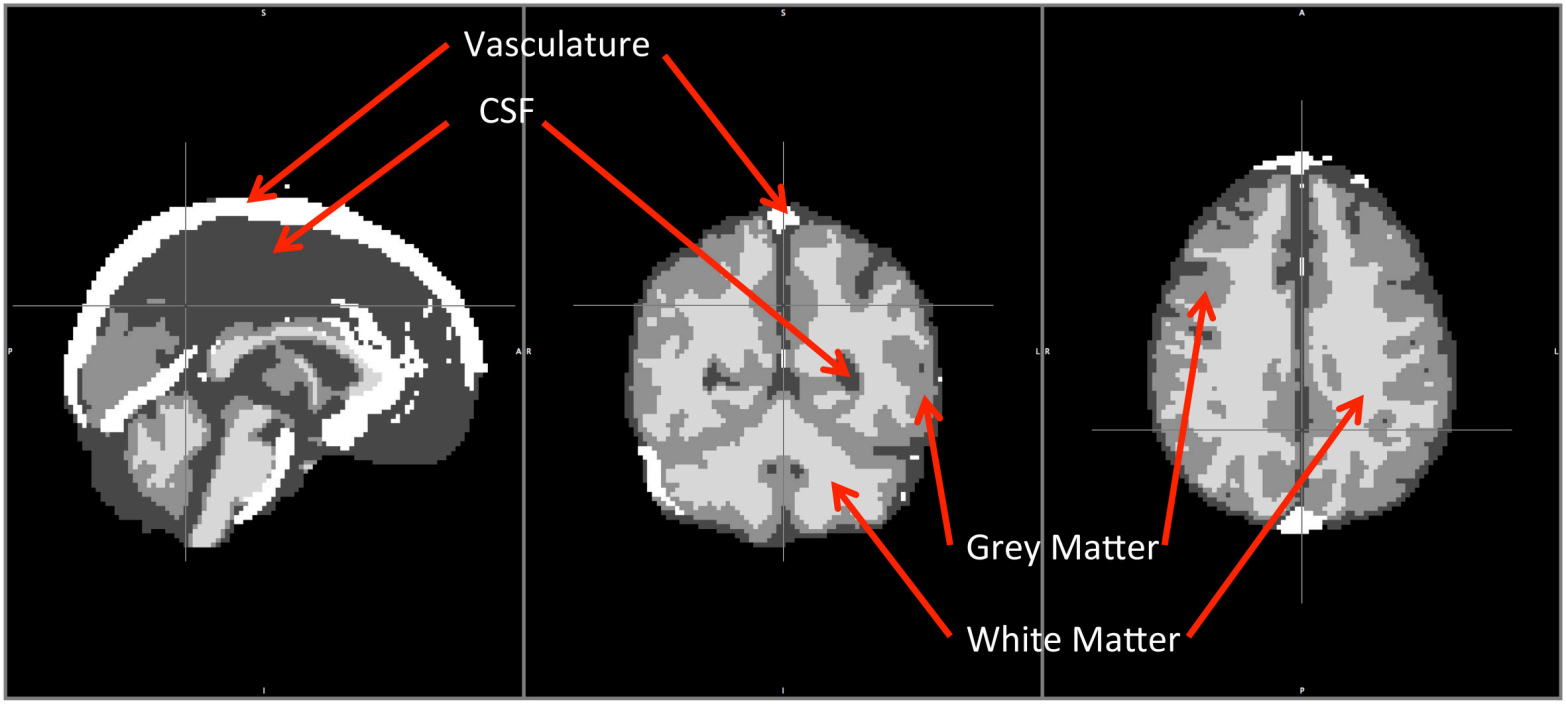
**Averaged segmentation of WM, GM, CSF and VA from 8 healthy participants**. WM, GM and VA were segmented by FAST. VA was extracted from the MRA scan (from 7 subjects).

All these segmented regions (WM, GM, CSF, and VA) later served as templates and masks for the study. The averaged segmented brain is shown in Figure 1, where WM, GM, CSF, and VA are displayed together.

### Maps of sLFO max-correlation coefficient (maxcc) and ALFF

**Figure 2:**
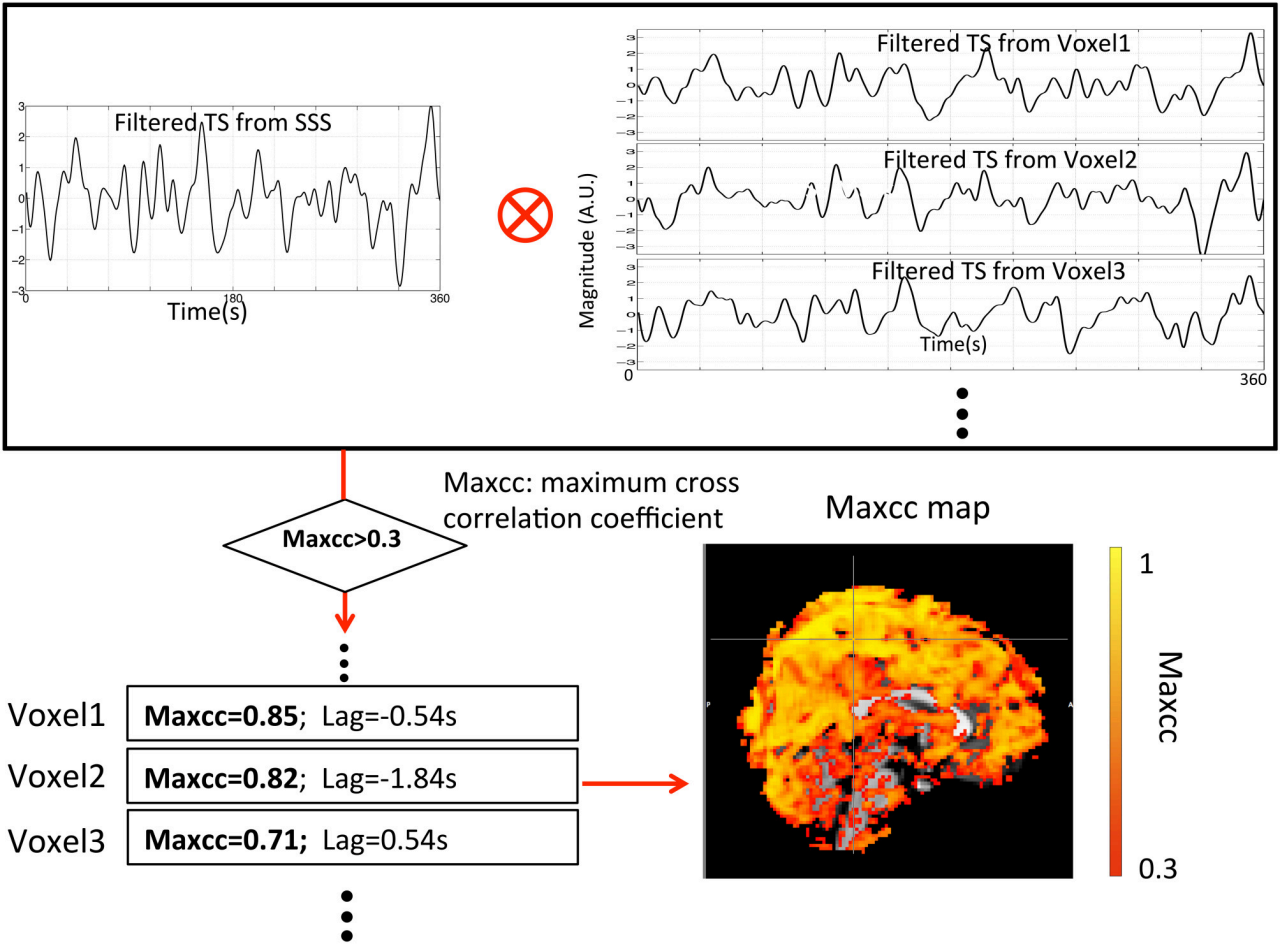
**Flowchart of the method used to calculate the maxcc map for each subject from the filtered RS data (0.01–0.15 Hz)**. The procedure started with the selection of the filtered seed regressor from the superior sagittal sinus (SSS) (mask). This regressor was then cross-correlated with all other BOLD signals to select the voxels that have significant maximum correlation coefficients (maxcc > 0.3). The corresponding maxcc of these voxels produce the colors on the maxcc map.

Two different maps (3D) were derived from each participant's resting state data: (1) a map of ALFF, in which the values represent the amplitudes of LFO from BOLD signal at each voxel, and (2) a map of the maximum cross-correlation (maxcc) of sLFO, in which the value of each voxel represents the best correlation between the optimally delayed sLFO extracted from the BOLD in Superior Sagittal Sinus (SSS) with the BOLD signal timecourse in every voxel. The details of the derivation are given in later sections and illustrated in Figure 2.

In order to create the ALFF map, we calculated the ALFF of preprocessed resting state data using the software package REST (Song et al., 2011). In brief, for each voxel, the Fourier transform was applied on preprocessed BOLD signal, and then the amplitude of the power spectrum (0.01–0.15 Hz) was summed.

In order to create the maxcc map, first a seed region of interest (ROI) was selected. Because we believe these sLFOs are related to the blood signal, the seed ROI was chosen from a section of a blood vessel (i.e., SSS) to avoid any contribution from neuronal activation. The seed ROI was hand-picked for each subject at the back of the brain with the help of MRA and FSL segmentation. The size of the ROI is similar among all the subjects and consists of voxels falling completely within the vessel. The averaged timecourse of the seed section was extracted and used to cross-correlate with all the BOLD signals from the rest of the voxels in the brain volume. After the cross-correlation, we selected voxels for further analysis that met the following conditions: (1) the maximum cross-correlation was significant (>0.3), and (2) the time lag of this maximum correlation was from −6 to +6 s. The cross-correlation search range was selected based upon our previous research (Tong and Frederick, 2014; Tong et al., 2016). As we have demonstrated (Tong et al., 2016), the range of actual delay times is far smaller than the search range of -6 to 6 s. However, since the lags between the seed regressor (extracted from the SSS) and the rest of the BOLD signals are subject-specific, the distribution of the lag values (from all the voxels) is never centered at zero. Having a larger range will cover most of the meaningful voxels regardless of the lags' distribution. As a result of choosing a wider correlation range, the chance of having spurious correlation problem increases. To minimize the effect of spurious correlation, the minimum correlation threshold was increased to 0.3, which compensates for the correlation-inflating effects of large cross-correlation range (time delay processing) and bandpass filtering (Davey et al., 2013; Hocke et al., 2016). The voxels that were selected by the procedure were called “valid” voxels, and were used in the following calculations. The procedure is depicted in Figure 2.

### Assessment of segmentation using different maps

**Figure 3:**
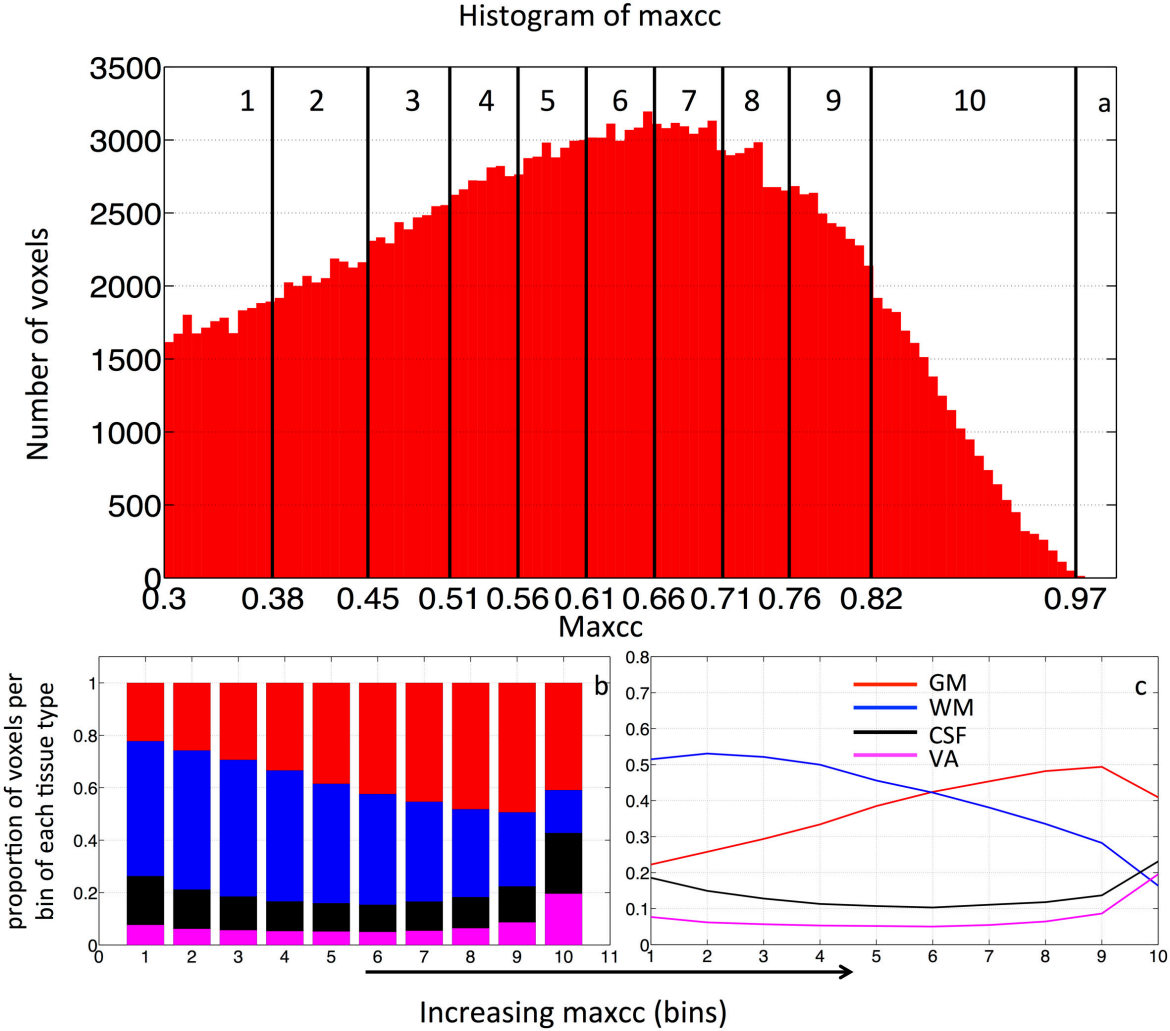
**(A)** Histogram of all the valid voxels in maxcc map of BOLD sLFO from one subject. The number of voxels with each maxcc value is graphed, yielding a distribution that can be divided into 10 bins (as marked by the black vertical lines), each containing 10% of the total number of voxels. The maxcc values that separate these 10 bins are shown on the X axis. For each bin, the percentage of voxels in GM (red), WM (blue) and CSF (black) and VA (magenta) were calculated using the masks shown in Figure 1, with stacked distribution by bin number showing the proportion of voxels per bin of each tissue type graphed in **(B)** and regular distribution of tissue type by bin graphed in **(C)**.

In order to test the hypothesis that voxels with low maximum-correlation values are more likely to be in WM (with minimal vascular density), whereas voxels with increasingly greater max-correlation values are correspondingly more likely to be located in the more-vascular GM or even in VA, we calculated the distribution of the voxels with ascending maxcc values among four brain regions (WM, GM, CSF, and VA). In detail: first, we calculated the maxcc map for each subject, then ranked all the valid voxels in ascending order based on their maxcc values. Next, we grouped the voxels into 10 bins, with an equal number of voxels in each bin (i.e., 10% of all valid voxels). We then calculated the percentage of voxels per bin located in the participant-specific WM, GM, CSF, and VA areas. The percent distributions of voxels in each bin are plotted in what we refer to as “distribution graph.” For the purpose of comparison, the same procedure was repeated on individual ALFF data. Lastly, the distribution graphs were averaged to assess the group effect. This procedure is depicted in Figure 3. The histogram of the maxcc map of BOLD sLFO from one subject is plotted in Figure 3A. The maxcc, shown on the x-axis, begins at 0.3, which is the minimum correlation threshold used to select the voxels for analysis. The black vertical lines in the histogram mark the edges of the 10 bins, spaced by the number of voxels (10%, roughly 18,000 voxels/bin). The corresponding distribution of each bin (into WM, GM, CSF, and VA) is shown in Figures 3B,C in both stacked form and the form of lines. The red, blue, black, and purple bars represent the proportion of the voxels in the WM, GM, CSF, and VA, respectively.

## Results

**Figure 4:**
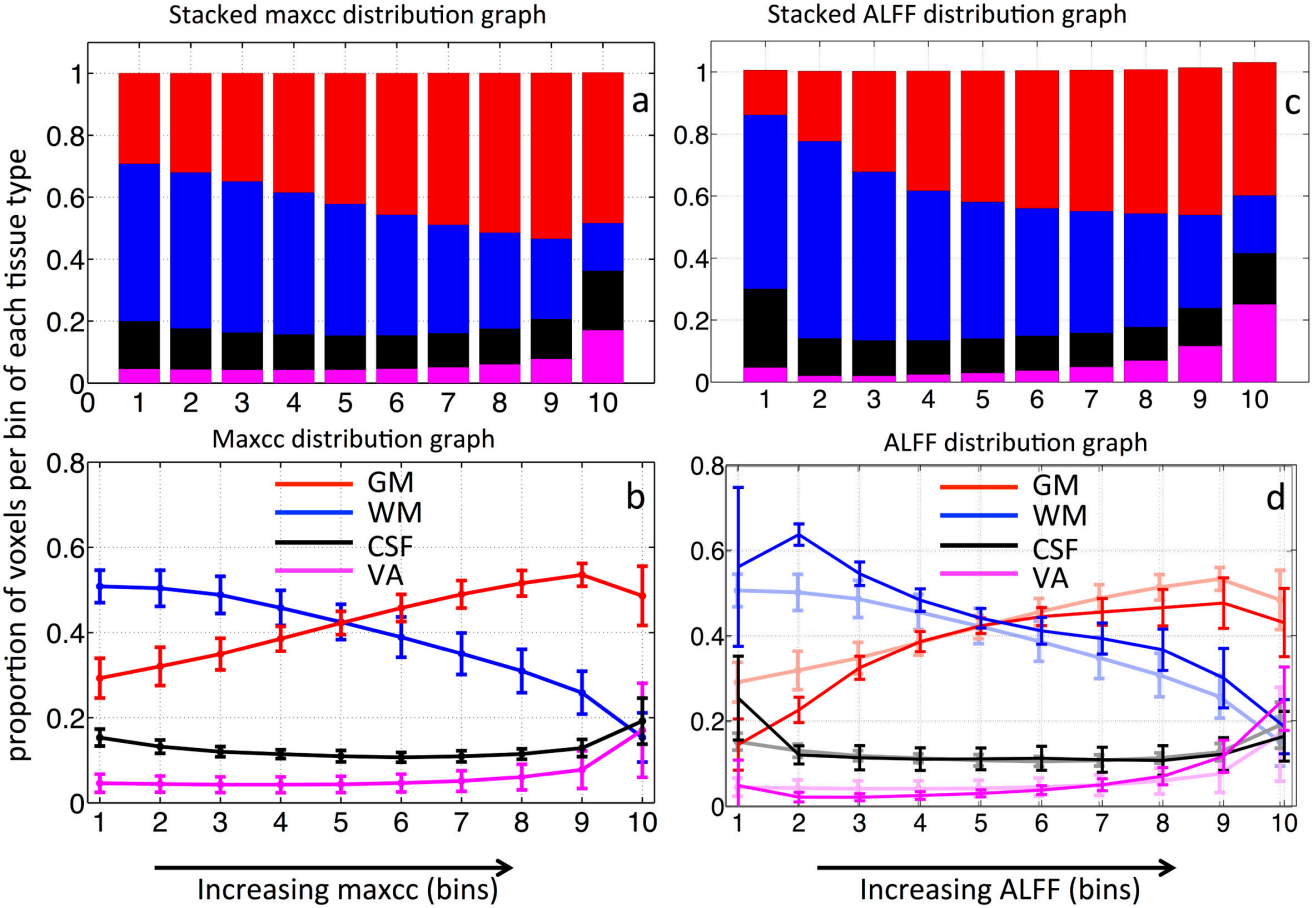
**Averaged stacked distribution graph of maxcc map in (A) and its corresponding regular distribution graph in (B)**. Averaged stacked distribution graph of ALFF map in **(C)** and its corresponding regular distribution graph in **(D)**. The regular distribution graph of averaged maxcc (as in **B**) is shown in the background of **(D)** as comparison. The error bars represent standard deviation.

Figure 4A shows the averaged stacked maxcc distribution graph from 8 subjects, where bars represent the tissue distribution of the voxels in each of the 10 bins. Different colors within the bar represent the proportion of these 10% voxels found in GM, WM, CSF, and VA respectively. The same averaged distribution graph with standard deviations is shown as Figure 4B. The maxcc distribution graph of each subject is shown in Figure S1. The distribution curves in GM, WM are highly consistent among all the subjects. When fitted with a linear model, the average slope of the curves in GM and WM are 0.028 ± 0.0077 and −0.031±0.012 respectively (Figures S1A,B). The curves in VA are also consistent, however, these data are poorly fit by a linear model.

From Figures 4A,B, we can see that as maxcc value increases, more voxels are found in GM. The opposite trend is observed for the case of WM. CSF and VA do not exhibit a strong relationship with maxcc except at the very highest correlation values (bin 10) where significantly more voxels are in these two tissue segments, compared to the values in bin 9 (*p* = 0.012 and *p* = 0.008 respectively from two sample *t*-test). As we know, CSF is not supposed to have any blood in it. However, the locations of CSF are next to those of GM and VA (see Figure 1). We believe the CSF results therefore largely represent segmentation and registration errors.

The stacked distribution graph of ALFF is shown in Figure 4C, with its non-stacked version shown in (d), overlaid upon the maxcc distributions from Figure 4B, which are shown as shaded lines in the background. Figure 4D shows the similarity between the distribution graph of ALFF and that of maxcc with small differences. The correlation coefficients of corresponding distribution curves are 0.97, 0.97, 0.65, 0.99 for GM, WM, CSF, and VA respectively). The differences are mostly found in bin 1 and 2, as ALFF value increases, the distribution curves become similar with that of maxcc.

**Figure 5:**
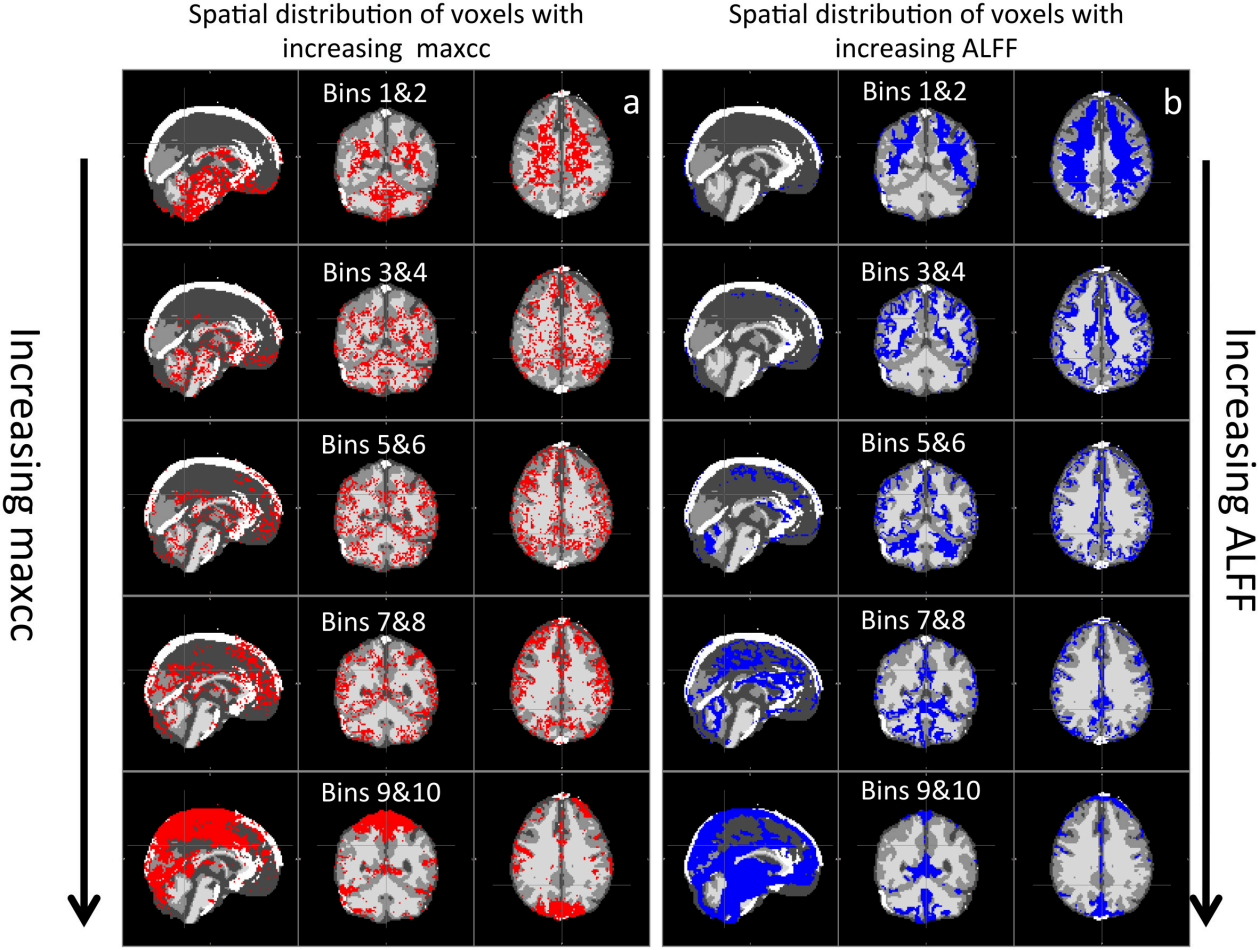
**Averaged spatial distribution of the voxels with increasing maxcc in (A) and increasing ALFF values in (B)**. Each panel represents the spatial distribution of the 20% of valid voxels (2 bins) ranked by increasing maxcc and ALFF values.

Figure 5 shows the spatial distributions of valid voxels corresponding to the increasing value in maxcc (a) and ALFF (b). For example, the first row of maps in Figure 5A represents the spatial distribution of the 20% of valid voxels that have the lowest maxcc values. The second row of maps represents the next 20% valid voxels with higher maxcc value and so on. From Figure 5, we can see the similarities in the following way: (1) the voxels that have the lowest values of maxcc and ALFF are clustered in WM as demonstrated in the first row of Figure 5; (2) As maxcc and ALFF values increase, the voxels are increasingly likely to appear in the GM areas; (3) the voxels with highest maxcc and ALFF are in large blood vessels as shown in the last row of Figure 5. The visible differences between the spatial distribution maps of maxcc and ALFF are: (1) the voxels with the lowest maxcc values can be found in the lower brain (e.g., pons), which is not true for that of ALFF; (2) the spatial patterns of maxcc are much noisier than those of ALFF, which have clearer boundaries; (3) even though voxels with highest maxcc and ALFF are in large blood vessels, the voxels with the highest maxcc values are clustered at the top and back of the brain (last map in Figure 5A), while the voxels with the highest ALFF values can be found in lower brain regions, near the pons (last map in Figure 5B).

## Discussion

This study demonstrates that tissue types significantly correlate with characteristics of the resting state BOLD signal, in both its amplitude and its temporal fluctuation. We have confirmed previous findings regarding BOLD ALFF. More importantly, we have revealed the relationship between the sLFO of BOLD signals and the tissue types in resting state. We demonstrated that the portion of the BOLD signal accounted for by these sLFOs in each voxel is positively correlated with the probability that these voxels are found in GM and VA, and negatively correlated with the probability that these voxels are found in WM. This finding suggests that the contribution of sLFOs to BOLD signal may be positively correlated with the voxel's underlying vascular density, which progressively decreases in these three tissue types: VA, GM, WM. The findings not only extend our knowledge of the parts (neuronal and non-neuronal) that compose the BOLD signal, but also indicate potential biomarkers for the assessment of vascular parameters, such as vascular density and symmetry. This knowledge is generally useful and, moreover, crucial for developing and evaluating denoising methods in resting state fMRI. However, denoising is not the focus of this manuscript. We have explored the issue in the previous studies (Frederick et al., 2012) and are currently working on the development of several novel methods.

Previously, great effort was expended to understand the time delays reflecting the relative arrival time of sLFOs in each voxel. Comparisons with Dynamic Susceptibility Contrast measurements, namely bolus tracking MR (Tong et al., 2016) have shown that the dynamic pattern of this sLFO moving through the brain is, to a large extent, related to blood flow. However, no previous studies thoroughly evaluated the correlation strengths of sLFO with BOLD signals. In this study, we demonstrate that correlation strengths between sLFO and BOLD signals are also meaningful. As shown in Figure 4A, the voxels with lower maxcc values are likely located in the tissues that have low blood density (WM), whereas voxels of higher maxcc values are more likely found in tissues that have higher blood density (e.g., GM and VA). Moreover, as shown in Figure 5, the spatial distributions of voxels with increasing maxcc values match those from ALFF, which has been shown to be closely associated with the blood density. Of greater interest, the specific manner in which more voxels are found in GM as maxcc and ALFF increases is clearly systematic. This is more obvious in the case of ALFF (Figure 5B), where the pattern with lowest ALFF value is at the center of the WM and, as ALFF increases, the corresponding voxels are found at the outer boundary of the previous pattern. These boundaries may represent contour lines of equal-blood-density. If this is in fact the case, the map of ALFF can be used to assess the integrity of the cerebral blood density, which may be altered by stroke or brain tumor. More studies are needed to clarify the issue. Similar spatial patterns can also be observed in Figure 5A, however, with much more noise, as maxcc is based upon correlation, where spurious correlation, even corrected, still has an effect. This would tend to decrease the SNR of the maxcc map, leading to speckled patterns. We assume that future studies with larger sample sizes will compensate for this.

In addition to low SNR in the maxcc map, there are some other clear differences between maps of maxcc and ALFF, which we believe are likely due to the sensitivities of the two methods. First, as in Figure 5, there are many voxels with high ALFF values clustered at the bottom of the brain—around the pons and medulla areas—that are not visible in the corresponding graph of maxcc. We believe this difference is mainly attributable to the fact that heartbeat is prominent in BOLD signals originating near arteries, which are located at the base of the brain (a T1 effect due to blood volume changes). Hence, the magnitudes of these BOLD signals are enlarged by the aliased pulsation signals, and the closer the signal origin is to the main arteries, the stronger the effect. This accounts for the disproportionately large magnitude seen in the bottom of the brain (where many arteries reside) in Figure 5B. However, these aliased signals are not sLFO. Moreover, since these voxels are located in or near the arteries, where there is little deoxy-hemoglobin (contrast in BOLD), the sLFO cannot be clearly detected. This explains why the voxels of these regions have the lowest maxcc values (top graph in Figure 5A). Second, the voxels are heavily clustered in the back of the head in the map of highest maxcc (last graph in Figure 5A), but not in the map of ALFF. This may be due to the fact that the seed of maxcc calculation was selected from the SSS from that region (Figure 2), leading to corresponding highest correlation values.

**Figure 6:**
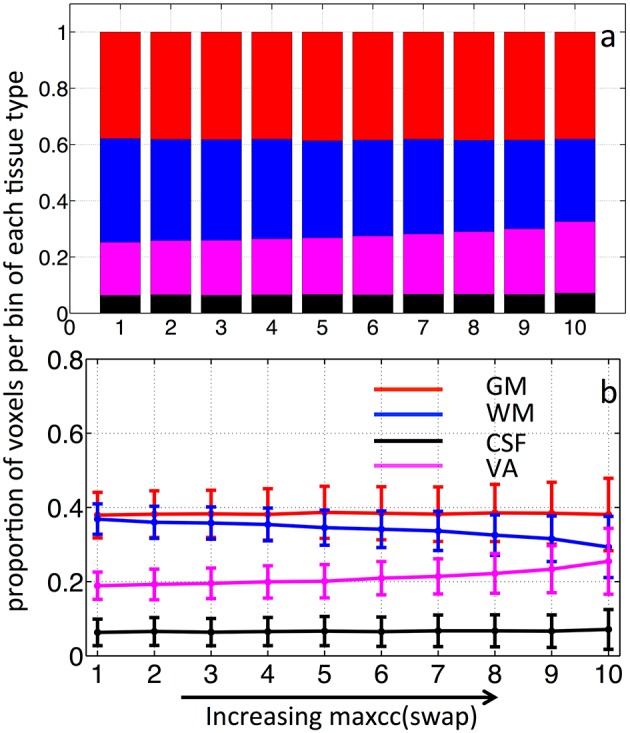
**Stacked distribution graph (A) and regular distribution graph (B) of the tissue distribution of voxels binned by averaged maxcc(swap) which was calculated from seed regressors that were swapped between subjects**.

As we know, the signal to noise ratio (SNR) of BOLD is likely to be greater in blood-rich tissues, regions with high density of capillaries and veins such as GM, and voxels containing large veins with high concentrations of deoxy-hemoglobin. In contrast, a tissue such as white matter has low SNR in BOLD for the opposite reason. It follows, then, that the SNR effect might bias the maxcc distribution toward GM and VA. In order to assess the degree of SNR influence, we recalculated the maxcc map for each subject. However, instead of using a subject's own seed timecourse measured within the SSS to calculate the maxcc, we used every other subject's seed timecourse (total of 56 swapped maxcc maps were calculated). With the seed regressors swapped in this manner, no meaningful maxcc value should be produced and the result should reflect the SNR effect only. The averaged result with standard deviation is shown in Figures 6A,B with stacked distribution and regular distribution graphs. From Figure 6, as the maxcc (swap) value increases from left to right in bins 1–10, there is no clear change in the number of voxels found in GM. The *t*-test confirms that no significant non-zero values were found in the slopes of the GM distribution curves (*p* = 0.77). This indicates that voxels are evenly distributed among all the tissue types regardless of the maxcc (swap) value, which further implies that SNR differences were not the main effects in real maxcc calculations in GM voxels. However, we did observe small changes in voxel distribution by bin in WM and VA, which means that SNR differences have small effects on the real maxcc calculations in WM and VA voxels. Lastly, we performed a two sample *t*-test between the slopes of the curves (GM and WM) in Figures 4B, 6B. They are significantly different (*p* = 4.5 × 10^−15^ and *p* = 4.6 × 10^−15^ respectively), which demonstrated that the effect we observed in Figure 4B can not be mainly due to SNR difference.

In this study, we have confirmed that the tissue type has significant influence on the ALFF of BOLD signals in resting state. More important, we have demonstrated that the portion of the BOLD signal accounted for by the sLFOs in each voxel is higly dependant on the tissue type. Since these tissue types significantly differ in vascular density, our results imply that the portion of the BOLD signal accounted for by the sLFOs, as well as ALFF value, in each voxel may be positively correlated with voxel's underlying vascular density. The limitation of the study is that MRA used in the study was not sensitive enough to separate vascular density within each tissue type, therefore we are not able to demonstrate the direct link between sLFOs/ALFF with vascular density accurately. The three main tissue types with different vascular densities were used as proxies in this study. The future studies will involve some MR method, such as susceptibility-weighted imaging (Descoteaux et al., 2008; Frangi et al., 2011), to assess the regional vascular density.

## Author contributions

YT performed image analysis, data interpretation, prepared original manuscript, and figures. BF designed and conducted experiments. LH, KL, GV, CC, and BF participated in data acquisition and quality control. SE helped in preparing the manuscript and figures. All participated in manuscript review.

## Funding

The work was supported by the National Institutes of Health, Grants K25 DA031769 (YT), R21 DA032746 (BF) and K08 DA037465 (GV). SE is supported by the Scientific and Technological Research Council of Turkey (TÜBITAK).

### Conflict of interest statement

The authors declare that the research was conducted in the absence of any commercial or financial relationships that could be construed as a potential conflict of interest.

## Supplementary material

Figure S1 **The maxcc distribution graph of 8 subjects is shown in (A) for GM, (B) for WM and (C) for VA**. Blue line is the averaged curves and purple line is the linear fitting of the curves.
