# Supplementary figures and images for: Systemic Low-Frequency Oscillations in BOLD Signal Vary with Tissue Type
Source: Front Neurosci. 2016 Jun 30;10:313. doi: 10.3389/fnins.2016.00313 (PMC4928460; doi:10.3389/fnins.2016.00313)

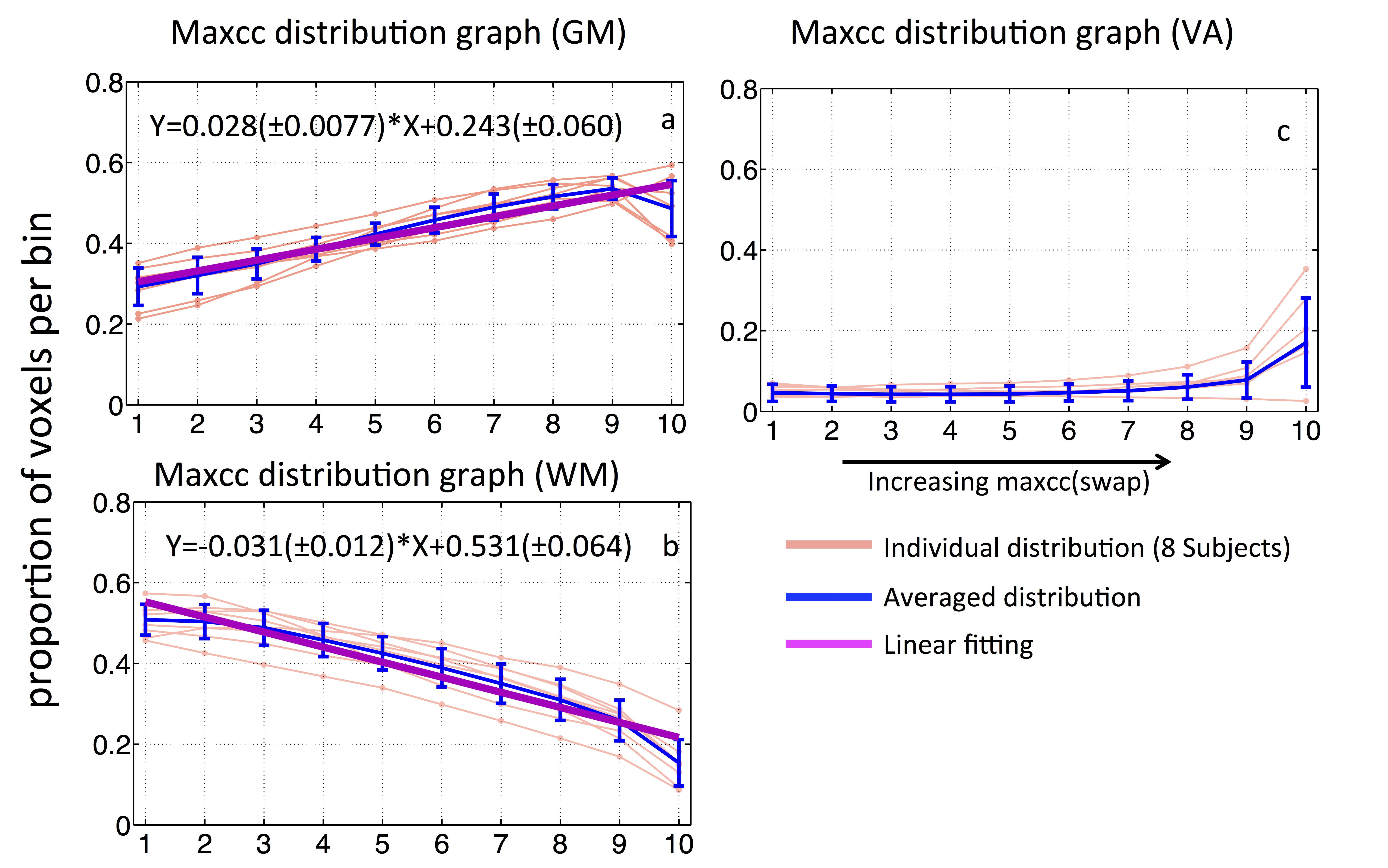

Supplement: Figure S1 — The maxcc distribution graph of 8 subjects is shown in (A) for GM, (B) for WM and (C) for VA. Blue line is the averaged curves and purple line is the linear fitting of the curves. [file Image1.JPEG]
